# Frozen elephant trunk technique using hybrid grafts: 15-year outcomes from a single-centre experience

**DOI:** 10.1093/ejcts/ezad364

**Published:** 2023-10-31

**Authors:** Giacomo Murana, Gregorio Gliozzi, Luca Di Marco, Francesco Campanini, Silvia Snaidero, Chiara Nocera, Paola Rucci, Giuseppe Barberio, Alessandro Leone, Luigi Lovato, Davide Pacini

**Affiliations:** Division of Cardiac Surgery, Cardio-Thoraco-Vascular Department, IRCCS Azienda Ospedaliero-Universitaria di Bologna, S.Orsola Hospital, University of Bologna, Bologna, Italy; Division of Cardiac Surgery, Cardio-Thoraco-Vascular Department, IRCCS Azienda Ospedaliero-Universitaria di Bologna, S.Orsola Hospital, University of Bologna, Bologna, Italy; Division of Cardiac Surgery, Cardio-Thoraco-Vascular Department, IRCCS Azienda Ospedaliero-Universitaria di Bologna, S.Orsola Hospital, University of Bologna, Bologna, Italy; Department of Experimental, Diagnostic and Specialty Medicine, DIMES, University of Bologna, Bologna, Italy; Division of Cardiac Surgery, Cardio-Thoraco-Vascular Department, IRCCS Azienda Ospedaliero-Universitaria di Bologna, S.Orsola Hospital, University of Bologna, Bologna, Italy; Division of Cardiac Surgery, Cardio-Thoraco-Vascular Department, IRCCS Azienda Ospedaliero-Universitaria di Bologna, S.Orsola Hospital, University of Bologna, Bologna, Italy; Division of Cardiac Surgery, Cardio-Thoraco-Vascular Department, IRCCS Azienda Ospedaliero-Universitaria di Bologna, S.Orsola Hospital, University of Bologna, Bologna, Italy; Department of Biomedical and Neuromotor Sciences, DIBINEM, University of Bologna, Bologna, Italy; Division of Cardiac Surgery, Cardio-Thoraco-Vascular Department, IRCCS Azienda Ospedaliero-Universitaria di Bologna, S.Orsola Hospital, University of Bologna, Bologna, Italy; Division of Cardiac Surgery, Cardio-Thoraco-Vascular Department, IRCCS Azienda Ospedaliero-Universitaria di Bologna, S.Orsola Hospital, University of Bologna, Bologna, Italy; Cardiovascular Radiology Unit, Department of Medical and Surgical Sciences (DIMEC), IRCCS Azienda Ospedaliero-Universitaria di Bologna, S.Orsola Hospital, University of Bologna, Bologna, Italy; Division of Cardiac Surgery, Cardio-Thoraco-Vascular Department, IRCCS Azienda Ospedaliero-Universitaria di Bologna, S.Orsola Hospital, University of Bologna, Bologna, Italy; Department of Experimental, Diagnostic and Specialty Medicine, DIMES, University of Bologna, Bologna, Italy

**Keywords:** Aortic arch surgery, Frozen elephant trunk, Aortic reintervention, Thoracic endovascular aortic repair

## Abstract

**OBJECTIVES:**

The purpose of the study is to compare the short- and long-term outcomes of the frozen elephant trunk (FET) technique based on 2 different hybrid grafts implanted from January 2007 to July 2022.

**METHODS:**

The study includes patients who underwent an elective or emergency FET procedure. Short-term, long-term mortality and freedom from thoracic endovascular aortic repair (TEVAR) were the primary end points. Analyses were carried out separately for the periods 2007–2012 and 2013–2022

**RESULTS:**

Of the 367 enrolled, 49.3% received E-Vita Open implantation and 50.7% received Thoraflex Hybrid implants. Overall mean age was 61 years [standard deviation (SD) = 11] and 80.7% were male. The average annual volume of FET procedures was 22.7 cases/year. Compared to E-Vita Open, patients implanted with Thoraflex Hybrid grafts were more likely to receive distal anastomosis in zone 2 (68.3% vs 11.6%, *P* < 0.001) with a shorter stent portion, mean = 103mm (SD = 11.3) vs mean = 149 mm (SD = 12.7; *P* < 0.001) and they underwent a reduced visceral ischaemia time, mean = 42.5 (SD = 14.2) vs mean= 61.0 (SD = 20.2) min, *P* < 0.001. In the period 2013–2022, overall survival at 1, 2 and 5 years was 74.8%, 72.5% and 63.2% for Thoraflex and 73.2%, 70.7% and 64.1% for E-Vita, without significant differences between groups (log-rank test = 0.01, *P* = 0.907). Overall freedom from TEVAR at 1, 2 and 5 years was 66.7%, 57.6% and 39.3% for Thoraflex and 79%, 69.7% and 66% for E-Vita, with significant differences between groups (log-rank test = 5.28, *P* = 0.029). In a competing risk analysis adjusted for chronic/residual aortic syndromes and stent diameter, the Thoraflex group was more likely to receive TEVAR during follow-up (subdistribution hazard ratio SHR = 2.12, 95% confidence interval 1.06–4.22).

**CONCLUSIONS:**

The FET technique addresses acute and chronic arch disease with acceptable morbidity and mortality. Downstream endovascular reinterventions are very common during follow-up.

**Figure:**
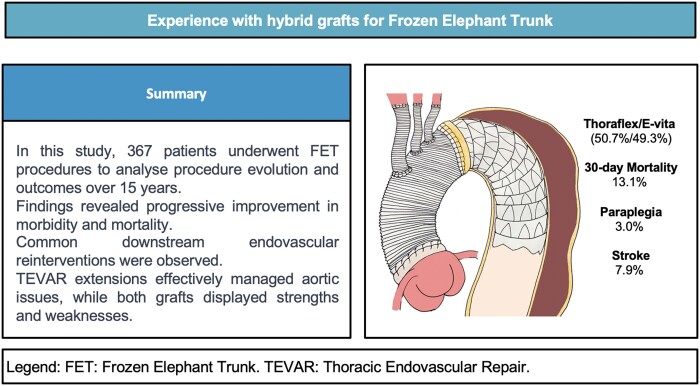

## INTRODUCTION

The frozen elephant trunk (FET) procedure is one of the recommended treatment options in cases of extensive arch pathologies [1]. According to the most recent consensus it should be considered in any scenario of proximal aortic repair where a secondary distal repair may be needed [1, 2]. Actual overall mortality ranges from 2.4% to 15.0%, overall stroke events are seen in 5.4–9.3%, renal replacement therapy is needed in 5–14.9% and spinal cord injury (SCI) rates range from 2% to 8.9% [3–5]. This large variability derives from patient selection, technique modifications and centre experience. Since its introduction in the early 2000s, the technique evolved and became more ‘surgeon-friendly’ due to proximalization of the distal anastomosis and left subclavian artery reimplantation using endografts [6–8]. Nowadays, the total number of FET procedures worldwide reaches about 11 000—a number expected to grow in the future according to the recent STS projections [9, 10].

The purpose of the study was to analyse the outcomes of our centre’s experience with the FET technique using the hybrid grafts available in Europe over a 15-year period.

## METHODS

### Ethics statement

Patients were identified through comprehensive quality registries at IRCCS Azienda Ospedaliero-Universitaria di Bologna. These data were approved for the use in human subject by the institutional review board (IRB No. 121/2022/Disp/AUOBo) that waived the need for written informed consent.

### Study design and patients

This retrospective study includes patients who underwent elective or emergency total arch replacement with FET procedure from January 2007 to July 2022.

The study involved patients with either chronic aortic disease or acute aortic syndromes (ASSs). Two different hybrid prostheses were implanted: to better understand the impact of different prostheses on patient outcomes, the cohort was divided into E-Vita and Thoraflex groups and into 2 period groups: 2007–2012 and 2013–2022 to consider the introduction of Thoraflex in 2013. Baseline features, intraoperative data and outcomes were reported and compared between groups. After the operation, patients were routinely followed up with computed tomography (CT) scan and clinical evaluation in our aortic outpatient clinic. Specifically, patients were routinely evaluated after 1, 3 and 12 months and yearly thereafter. Clinical information were retrieved through medical charts, direct examinations and proprietary datasets.

Early (30 days), long-term (5 years) mortality and freedom from thoracic endovascular aortic repair (TEVAR) were the primary end points.

### Surgical technique

Our indications and surgical technique for FET procedure have been previously described [11, 12]. Briefly, the main features of our current standard approach are summarized below:

1. Antegrade arterial perfusion as first choice (in order of preference axillary, innominate and carotid artery);
2. moderate hypothermia with a target nasopharyngeal temperature of 25–26°C;
3. antegrade selective cerebral perfusion (ASCP) with 10–15 ml/kg/min; and
4. spinal cord protection with cerebrospinal fluid (CSF) drain in all elective cases, without any contraindications.

Our surgical technique has been modified and standardized over time to improve outcomes and to extend the indications of FET technique both in elective and in emergent settings. The most relevant advance was a more proximal implantation of the prosthesis in arch zone 2. In particular, with the E-Vita prosthesis anastomosis was performed in zone 2 (21, 11.6%)—zone 3 (160, 88.4%) and with the Thoraflex prosthesis in zone 2 (127, 68.3%)—zone 3 (59, 31.7%).

**Figure 1:**
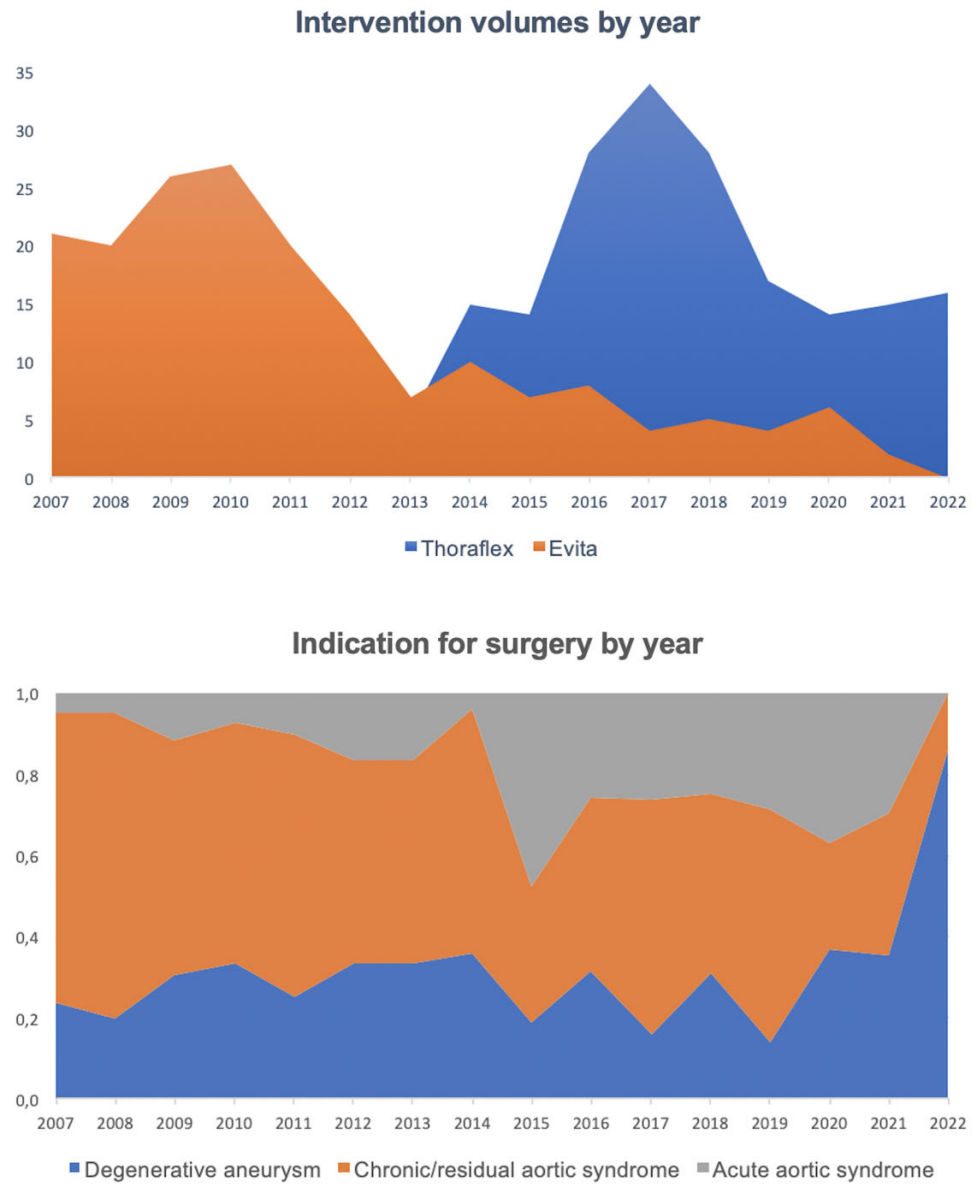
Annual volume of frozen elephant trunk implantations divided by prosthesis brand and indication for surgery.

The evolution on hybrid graft selection is reported in Fig. 1: at the beginning of the series, the E-Vita Open (JOTEC GmbH, Hechingen, Germany) prosthesis was the only prosthesis available. Since 2013 we adopted routine use of the Thoraflex Hybrid Plexus prosthesis (Terumo Aortic, Vascutek Ltd, Ichinnan, UK). More recently, prosthesis selection has been in favour of the Thoraflex Plexus because it facilitates an anatomical reconstruction of the epiaortic vessels allowing a fast distal reperfusion through the side branch after the circulatory arrest. E-Vita was used in hostile arch lesions where a longer stent with a stronger radial force could have been curative.

The method of size selection of the prostheses was similar and based on Angio-CT evaluation. Preoperative planning is performed using multiplanar reconstruction CT images. In the sagittal plane, starting from zone 2 (according to Ishimaru aortic map) we estimate the site of the distal landing zone of the stent graft. Then, we move to the axial plane to measure the maximum true lumen length in case of aortic dissections or the entire lumen diameter for aneurysms. In the acute setting, we do not perform any oversizing, while in chronic aneurysms we oversize by 20%.

### Statistical analysis

Categorical variables were summarized as absolute and percentage frequencies, while continuous variables were summarized as mean and standard deviation (SD). Variables were compared between groups using chi-squared test, Fisher’s exact test, Student’s *t*-test or Mann–Whitney test, as appropriate. Survival was estimated with Kaplan–Meier curves, and groups were compared with the log-rank test. Binary logistic and proportional hazard Cox regressions were performed to compare early and long-term mortality between E-Vita and Thoraflex prostheses in the period 2013–2022. Propensity score adjustment was used to control for confounding. Factors that could influence the outcomes were used to generate a propensity score by logistic regression. The variables used included age, gender, degenerative aneurysm and chronic/residual aortic syndrome (AS).

Risk for aortic reoperation (TEVAR) was analysed through the multivariable Fine and Gray competing risk model (1999), considering death as a competing event, with the Stata procedure stcrreg. The time trend of in-hospital and 1-year mortality was investigated using restricted cubic splines.

Statistical analysis was performed using IBM SPSS Statistics, version 26.0 (IBM Corp, Armonk, NY, USA) and STATA 17 (StataCorp LLC, College Station, TX, USA).

## RESULTS

### Baseline features

Our overall experience includes 181 (49.3%) E-Vita implants, of which 128 implanted in 2007–2012 and 53 in 2013–2022, and 186 (50.7%) Thoraflex hybrid implants. The average annual volume of FET procedures was 22.7 cases/year (Fig. 1). The main indications for surgery were chronic/residual AS in 54.8%, degenerative aneurysm in 26.4%, AAS in 17.4% and pseudoaneurysm in 1.4% of cases: starting from 2015 the use of FET procedure in AAS was more frequent (8.5% during the first period versus 25.2% during the second half of experience, *P* < 0.001).

Baseline features are reported in Table 1. The mean age was 61 (SD = 11) years and 296 (80.7%) were male. E-Vita and Thoraflex populations differed significantly in the proportion of male gender (85.6% vs 75.8%, *P* = 0.017), indication for surgery (degenerative aneurysm 34.3% vs 18.8%, chronic/residual AS 52.5% vs 57%, AAS 12.2% vs 22.6%, pseudoaneurysms 1.1% vs 1.6%; *P* = 0.002) and preoperative cerebral malperfusion (1.7% vs 5.9%; *P* = 0.032).

**Table 1:** Population characteristics

Baseline features	Overall, *n* = 367	E-Vita, *n* = 181	Thoraflex, *n* = 186	*P*-value
Age (years), mean (SD)	61 (11)	61 (11)	61 (12)	0.744
BMI (kg/m^2^), mean ± SD	26.7 ± 4.5	26.8 ± 4.0	26.5 ± 4.8	0.487
Gender, male, *n* (%)	296 (80.7)	155 (85.6)	141 (75.8)	**0.017**
Hypertension, *n* (%)	305 (83.3)	153 (84.5)	152 (82.2)	0.543
Smoking, *n* (%)	162 (44.3)	87 (48.1)	75 (40.5)	0.147
Diabetes, *n* (%)	18 (4.9)	5 (2.8)	13 (7.0)	0.059
CAD, *n* (%)	32 (8.7)	20 (11.0)	12 (6.5)	0.122
COPD, *n* (%)	40 (10.9)	25 (13.8)	15 (8.1)	0.080
CKD, *n* (%)	47 (12.8)	27 (14.9)	20 (10.8)	0.240
Cerebro-vascular disease, *n* (%)	17 (4.6)	8 (4.4)	9 (4.9)	0.840
Indication for surgery, *n* (%)				**0.002**
Degenerative aneurysm	97 (26.4)	62 (34.3)	35 (18.8)	
Chronic/residual aortic syndrome	201 (54.8)	95 (52.5)	106 (57.0)	
Acute aortic syndrome	64 (17.4)	22 (12.2)	42 (22.6)	
Type A	46 (12.6)	16 (8.9)	30 (16.1)	
Type B	18 (4.9)	6 (3.3)	12 (6.5)	
Pseudoaneurysm	5 (1.4)	2 (1.1)	3 (1.6)	
Marfan syndrome	26 (7.1)	14 (7.7)	12 (6.5)	0.642
Redo	192 (54.0)	95 (52.5)	103 (55.4)	0.579
Malperfusion	43 (11.8)	17 (9.4)	26 (14.1)	0.172
Cerebral	14 (3.8)	3 (1.7)	11 (5.9)	**0.032**
Spinal cord	11 (3.0)	5 (2.8)	6 (3.2)	0.795
Bowel	6 (1.6)	2 (1.1)	4 (2.2)	0.426
Renal	24 (6.6)	11 (6.1)	13 (7.0)	0.724
Lower limb	8 (2.2)	2 (1.1)	6 (3.2)	0.162

*P*-value <0.05 was considered statistically significative. Values in bold indicate statistical significance.

BMI: body mass index; CAD: coronary artery disease; CKD: chronic kidney disease; COPD: chronic obstructive pulmonary disease; SD: standard deviation.

Table 2 reports the operative features: emergency surgery was performed more frequently in the Thoraflex group (31.7% vs 20.2%, *P* = 0.013). In the vast majority of cases, the innominate or axillary artery was preferred as cannulation site (32.4% and 29.4%, respectively), with significant differences between groups. The mean duration of overall cardiopulmonary bypass (CPB) and myocardial ischaemia was 230 (SD = 68) and 145 (SD = 52) min, respectively. Although the mean CPB, mean = 232 (SD = 62) vs 230 (SD = 73), *P* = 0.740, and cross-clamp time mean = 149 (SD = 48) vs 141 (SD = 56), *P* = 0.121, were similar in the 2 groups, the mean duration of ASCP time was significantly longer, 103 (SD = 41.2) vs 90.1 (SD = 32.0) min, *P* = 0.001 and the mean visceral ischaemia was shorter 42.5 (SD = 14.0) vs 61.0 (SD = 20.2) min, *P* < 0.001, in the Thoraflex group. Regarding the surgical technique, the majority of patient who received a Thoraflex prosthesis got a distal anastomosis in zone 2 (68.3% vs 11.6%, *P* < 0.001) and separate rerouting of the epiaortic vessels (100% vs 44.8%, *P* < 0.001); moreover, the mean stent length was significantly shorter, Thoraflex 102.7 (SD = 11.3) vs 148.7 (SD = 12.7), *P* < 0.001, and with a shorter mean diameter, Thoraflex 30 mm (SD = 4.3) vs 31.6 (SD = 4.6) mm, *P* = 0.004.

**Table 2:** Operative features

Operative features	Overall (*n* = 367), *n* (%)	E-Vita (*n* = 181), *n* (%)	Thoraflex (*n* = 186), *n* (%)	*P*-value
Emergency	95 (26.1)	36 (20.2)	59 (31.7)	**0.013**
Arterial cannulation				
Innominate	119 (32.4)	55 (30.4)	64 (34.4)	0.411
Axillary	108 (29.4)	78 (43.1)	30 (16.1)	**<0.001**
Femoral	55 (15.0)	21 (11.6)	34 (18.3)	0.073
ascending/arch	55 (15.0)	23 (12.7)	32 (17.2)	0.228
Carotid	30 (8.2)	4 (2.2)	26 (14.0)	**<0.001**
CSF drain	169 (46.2)	84 (46.4)	85 (45.9)	0.929
Concomitant procedures	164 (44.7)	78 (43.1)	86 (46.2)	0.545
Bentall	83 (22.6)	34 (18.8)	49 (26.3)	0.084
David	8 (2.2)	0 (–)	8 (4.3)	**0.004**
AV replacement/repair	41 (11.2)	30 (16.6)	11 (5.9)	**<0.001**
MV replacement	4 (1.1)	2 (1.1)	2 (1.1)	0.678
CABG	34 (9.3)	18 (9.9)	16 (8.6)	0.657
CPB bypass time (min), mean ± SD	230 ± 68	232 ± 62	230 ± 73	0.740
Cross-clamp time (min), mean ± SD	145 ± 52	149 ± 48	141 ± 56	0.121
ASCP time (min), mean ± SD	97 ± 37	90 ± 31	103 ± 41	**0.001**
Visceral ischaemia time (min), mean ± SD	52 ± 20	61 ± 20	43 ± 14	**<0.001**
Zone 2 anastomosis	148 (40.3)	21 (11.6)	127 (68.3)	**<0.001**
Separate reimplantation of EV	266 (72.5)	81 (44.8)	186 (100)	**<0.001**
Stent length (mm), mean ± SD	125 ± 25	149 ± 13	103 ± 11	**<0.001**
Stent diameter (mm), mean ± SD	31 ± 5	32 ± 5	30 ± 4	**0.004**

*P*-value <0.05 was considered statistically significative. Values in bold indicate statistical significance.

ASCP: antegrade selective cerebral perfusion; AV: aortic valve; CABG: coronary artery bypass grafting; CPB: cardiopulmonary bypass; CSF: cerebrospinal fluid; EV: epiaortic vessels; MV: mitral valve; SD: standard deviation.

### Early outcomes

**Figure 2:**
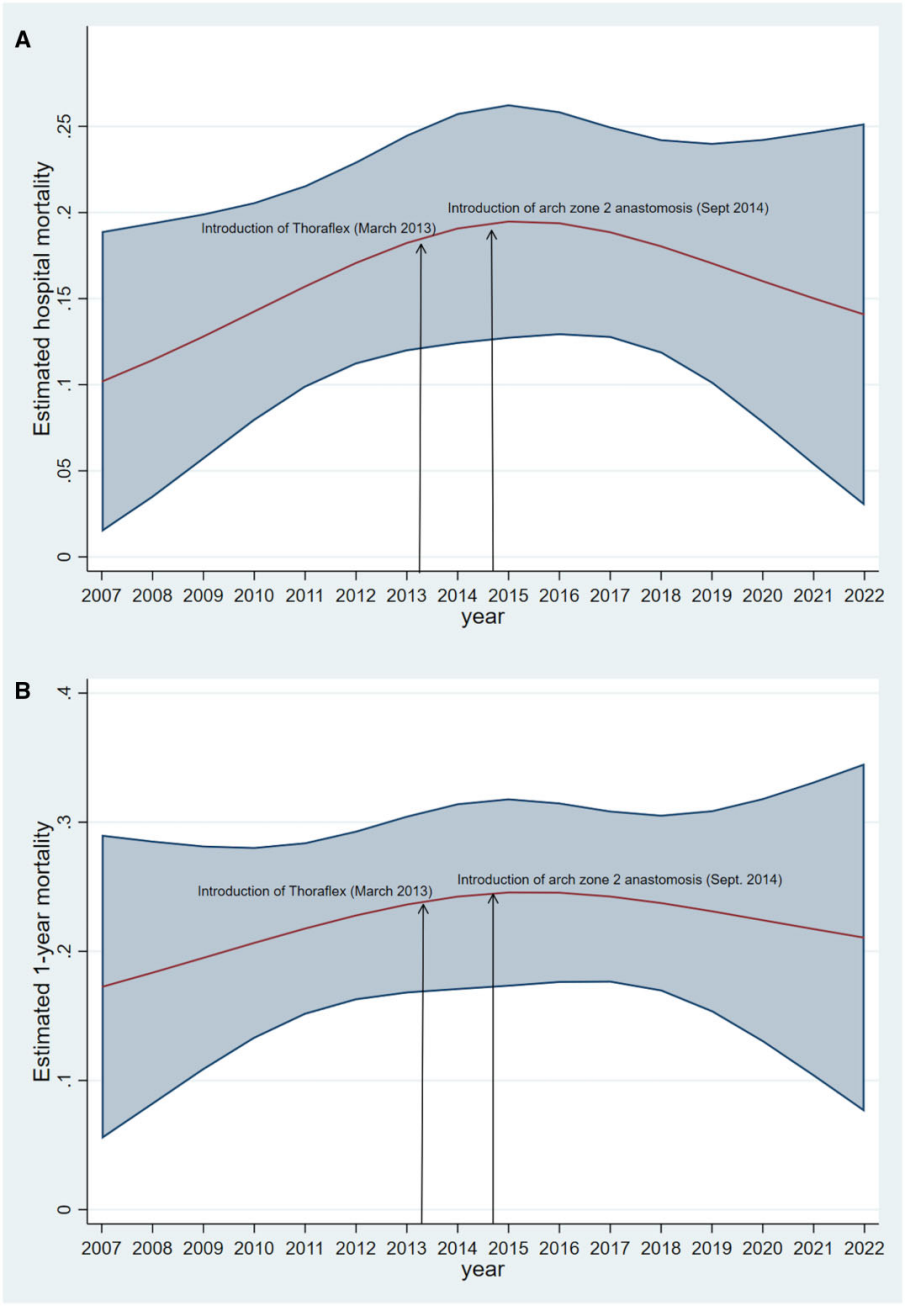
Trend of in-hospital (**A**) and 1-year (**B**) mortality in the overall sample in the years 2007–2022.

From 2007 to 2022, in-hospital mortality per year ranged between 10% and 18%. Figure 2A shows that the estimated trend of in-hospital mortality was non-linear, with a peak in the year following the introduction of Thoraflex and the extension of FET surgery to patients with aortic dissection. As regards other early outcomes (Table 3), patients treated with a Thoraflex prosthesis had a lower ventilation time, median = 18 (Inter-quartile range IQR 10–44.7) vs median = 20 (IQR 14.77) h, *P* = 0.017, lower rates of permanent new dialysis (8.6% vs 15.5%, *P* = 0.045) and paraparesis (2.7% vs 8.8%, *P* = 0.012). In 1 case, paraplegia was caused by a spinal dural haematoma after removal of the CSF drainage. Hoarseness of voice was observed with similar rates in zone 3 (21/219, 9.6%) and zone 2 (20/148, 13.5%) distal anastomosis site groups.

**Table 3:** Early outcomes

Early outcomes	Overall	E-Vita	Thoraflex	*P*-value
	(*n* = 367), *n* (%)	(*n* = 181), *n* (%)	(*n* = 186), *n* (%)	
ECLS	9 (2.5)	3 (1.7)	6 (3.2)	0.331
Re-sternotomy for bleeding	48 (13.1)	22 (12.2)	26 (14.1)	0.590
Ventilation time (h), mean ± SD	83 ± 190	106 ± 225	61 ± 147	**0.025**
Tracheostomy	37 (10.1)	20 (11.0)	17 (9.1)	0.543
Permanent dialysis	44 (12.0)	28 (15.5)	16 (8.6)	**0.045**
Paraparesis	21 (5.7)	16 (8.8)	5 (2.7)	**0.012**
Paraplegia	11 (3.0)	8 (4.4)	3 (1.6)	0.117
Stroke	29 (7.9)	15 (8.3)	14 (7.5)	0.787
ICU stay (days), mean ± SD	10 ± 17	11 ± 19	8 ± 13	0.108
Hospital stay (days), mean ± SD	23 ± 21	21 ± 18	25 ± 24	0.102
30-Day mortality	48 (13.1)	20 (11.0)	28 (15.1)	0.255
Degenerative aneurysm	14 (14.4)	9 (14.5)	5 (14.3)	**0.001**
Chronic/residual aortic syndrome	18 (9.0)	6 (6.3)	12 (11.3)	
Acute aortic syndrome	16 (25.0)	5 (22.7)	11 (26.2)	

The significance level was set at *P* < 0.05. Values in bold indicate statistical significance.

ECLS: extracorporeal life support; ICU: intensive care unit.

Overall mortality at 30 and 90 days was 13.1% and 18%, respectively, with no significant differences between groups.

### Late outcomes

The median follow-up was 27 months (IQR = 3–59) for Thoraflex and 67 months (IQR 11–132) for E-Vita. The estimated 1-year outcome increased slightly up to year 2015 and then started to decline (Fig. 2B).

**Figure 3:**
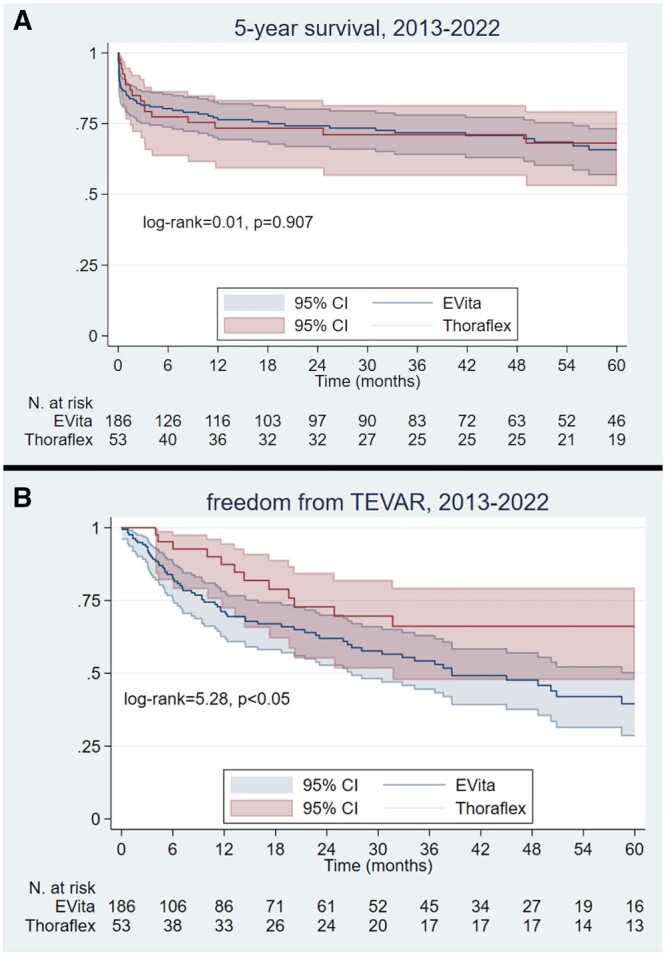
Five-year survival (**A**) and freedom from thoracic endovascular aortic repair (**B**) of E-Vita and Thoraflex groups in the years 2013–2022.

Five-year survival and freedom from TEVAR were analysed for the period between 2013 and 2022, in which both prostheses were used (Fig. 3) and compared between the period 2007–2012 and 2013–2022 for E-Vita.

Overall survival at 1, 2 and 5 years was 74.8%, 72.5% and 63.2% for Thoraflex and 73.2%, 70.7% and 64.1% for E-Vita, without significant differences between groups (log-rank test = 0.01, *P* = 0.907) (Fig. 3A).

After adjusting for the propensity score, the HR of 5-year mortality for E-Vita was 1.04, 95% confidence interval 0.57–1.90, *P* = 0.890.

The vast majority of patients requiring distal reintervention at follow-up underwent endovascular procedures; only 3 patients underwent thoraco-abdominal aortic aneurysm repair (open or hybrid).

The main indications for downstream TEVAR were progressive dilatation, residual post-dissecting aneurysms, distal stent-induced new entry tear (dSINE) and endoleaks (type Ib, II or III). In particular, dSINE was observed to be a more recent complication (*n* = 54), present since the introduction of Thoraflex zone 2 anastomosis (15.5%).

Overall freedom from TEVAR (Fig. 3B) at 1, 2 and 5 years was 66.7%, 57.6% and 39.3% for Thoraflex and 79%, 69.7% and 66% for E-Vita, with significant differences between groups (log-rank test = 5.28, *P* = 0.03).

**Figure 4:**
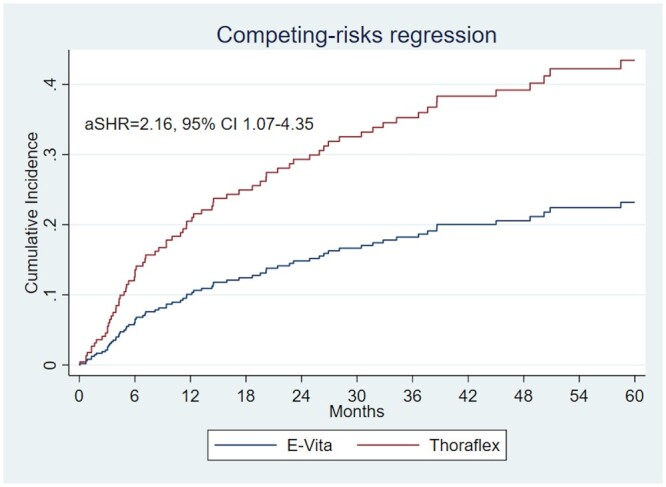
Competing risk regression for aortic endovascular reintervention after frozen elephant trunk according to hybrid graft (with death as a competing event) and adjusted for chronic/residual aortic syndrome and stent diameter for the years 2013–2022.

In a competing risk analysis (Fig. 4) with death as a competing event and adjusted for the propensity score and for the stent diameter, patients with a Thoraflex prosthesis were significantly more likely to undergo TEVAR over 5 years in the period 2013–2022 (aSHR = 2.16, 95% confidence interval 1.07–4.35).

Lastly, we compared the outcomes of patients with E-Vita implant operated in 2007–2012 and 2013–2022. The 5-year survival and the freedom from TEVAR in patients with E-Vita implants did not differ significantly between the 2 periods (see Supplementary Material, Figs S1 and S2).

## DISCUSSION

The ‘earthquake’ caused by the new transcatheter approaches to aortic disease has changed the way we look at surgery for aortic disease. The FET technique changed the paradigm of aortic arch treatment, introducing endovascular procedures as part of open surgery for the first time. It allowed surgeons to become more familiar with endovascular technologies and introduced hybrid techniques as part of the surgical armamentarium.

This study seamlessly demonstrates how the use of the FET has changed the treatment of thoracic and thoraco-abdominal aortic disease.

Our experience in aortic arch surgery began in the early 1970s with Professor Pierangeli, when he began to routinely use deep hypothermic circulatory arrest combined with ASCP to effectively protect the brain during arch replacement procedures [13]. Since then, >1000 arch operations have been performed using these 2 surgical hallmarks of cerebral protection [14]. However, the third milestone in the history of our centre was the introduction of FET in 2007. To date, this technique has been used in almost 400 cases and has become our preferred approach for arch replacement, making our centre the largest in Europe for implantation of both E-Vita Open and Thoraflex hybrid grafts.

The 3 main findings of this study can be summarized as follows:

1. Survival after FET is similar to that described in the literature for conventional arch replacement (76% at 1 year and 66% at 5 years); however, the use of a hybrid graft facilitates subsequent endovascular interventions by providing an easy proximal landing zone. It may also be curative in selected cases of AAS with arch or proximal descending thoracic aorta tears that do not require further intervention. The ability to cover a distal entry tear and early re-expansion of the true lumen allows early thrombosis of the false lumen and facilitates chronicity of the disease.
2. SCI has decreased over time, the lowest rate (<3%) reported with the shorter 100mm Thoraflex Hybrid stent graft. A more proximal zone 2 arch anastomosis has also been important in reducing this complication.
3. The long follow-up available in this study showed that aortic re-interventions after FET implantation are very common and are often used to treat residual distal aortic disease or new stent graft-related complications. They are most commonly observed within the first 36 months after the procedure and are usually easily managed with planned completion procedures.

In our experience, the most common indications for FET were residual AS in 54.8% of cases and degenerative aneurysm in 26.4% of cases. However, we have seen a rapid increase in the use of this treatment for AAS. In-hospital mortality remained fairly stable over the years at an acceptable rate of 13.1%, which was similar for the 2 hybrid grafts. This also explains the high rate of redo (54.0%) and concomitant procedures (44.7%). On the other hand, we observed a large variability in mortality rates depending on the underlying aortic pathology, ranging from 9% in elective repair of residual aortic dissection to 25% in acute type A aortic dissection. The high mortality rate reported in acutely treated patients was strongly influenced by the initial decision to only use FET in patients with severe organ malperfusion, including stroke and signs of mesenteric ischaemia. Over the past 3 years, we have seen a reduction in early mortality in these groups of patients with arch tear or rupture, as FET has been increasingly used in patients with only radiological—rather than clinical—signs of distal malperfusion.

In 2018, we shared our experience with the Hannover group and compared 437 patients undergoing FET with hybrid prostheses in a similar study [15].

Overall rates of postoperative complications were higher compared to previous results, including an overall in-hospital mortality of 14.9%, 10.8% of permanent neurological deficit and 5.5% of SCI. A total of 23% required an additional procedure, 16.3% required endovascular extension and 6.7% required aortic surgery [15]. However, the study suffered from major limitations: variability stemming from 2 different centres and approaches (from stent graft sizing to patient selection, surgical technique, postoperative practices, etc.) and a very short follow-up (median 2.6 years).

Technique-wise, we maintained the same approach to arch replacement throughout the entire study period. Antegrade arterial perfusion via the axillary, innominate or carotid arteries was our preferred site for CPB and ASCP. Moderate hypothermic circulatory arrest with a target nasopharyngeal temperature of 25–26°C was always preferred. A flexible endoscope was routinely used to explore the descending thoracic aorta before and after stent graft release to verify the positioning of the FET. Anastomotic sequences were maintained and we always preferred to directly reimplant the left or aberrant right subclavian artery in an anatomical repair [16, 17]. On the other hand, 2 major advances in the technique were the introduction of the trifurcated graft in 2013 and the arch zone 2 anastomosis in 2014. Since then, the branched Thoraflex Hybrid has become our preferred graft, as shown in Fig. 1. The device is easier to manoeuvre and it also has a 100-mm stent graft length and a dedicated side branch for distal reperfusion that allowed us to reduce operative times and risk of paraplegia. Following the improvements in our technique, the group of patients who received a trifurcated hybrid graft reported a lower ventilation time (61 vs 106 h, *P* = 0.025) and a lower rate of permanent new dialysis (8.6% vs 15.5%, *P* = 0.045). However, the recent introduction of the new E-Vita Open Neo from Artivion with a new trifurcated configuration and an easy delivery system is expected to fill this gap.

The other important aspect of this procedure was SCI. In our experience, the rate of paraplegia was 4.3% and we observed a significant reduction in this complication after performing a more proximal zone 2 arch anastomosis and with the introduction of the Thoraflex hybrid. However, to avoid this complication, we prefer to place a preoperative CSF drainage tube to monitor intracranial pressure in high-risk patients. Unfortunately, this manoeuvre itself can sometimes cause a spinal epidural haematoma after removal and was the cause of paraplegia in 1 patient.

Important conclusions can be drawn by comparing the results of our single-centre experience with the international E-Vita Open registry, which reports the largest European experience with FET in 1165 patients from 19 centres [5]. Tsagakis *et al.* [5] stratified the data according to the evolution of FET treatment over time: the first period, 2005–2011, was compared with the second period, 2012–2018. In the second period, bilateral selective cerebral perfusion remained the most common technique for cerebral protection, more ascending aorta and aortic valve sparing procedures were performed and visceral ischaemia times decreased significantly. However, despite the significant decrease in cerebral complications over time (10% vs 6%), the rate of permanent SCI-related symptoms remained similar (8% vs 6%) [5].

Compared to our lower incidence of paraplegia, the explanation for the higher rate of spinal complications may be found in a longer coverage of the descending thoracic aorta with stent graft lengths from 150 to 130 mm. Similarly, higher rates of paraplegia (7%) have been reported by the Hannover group using only the Thoraflex Hybrid [18]. Again, a higher rate of 150-mm grafts has been described, including the use of this technique in aortic dissections [18]. To reduce this type of complication, we always prefer to use a 100-mm stent graft length, if not in exceptional, selected cases where FET can be curative in a single-stage procedure. In addition, we are aware that these patients would certainly require a second procedure for endovascular completion, and therefore our policy is to stage thoracic or thoraco-abdominal treatments to facilitate preconditioning of the spinal cord supply.

The strength of this study is the availability of long-term results with both grafts. We observed a similar survival rate between E-Vita and Thoraflex patients, with a 5-year survival rate of 67% and 66%, respectively (log-rank test, *P* = 0.973). These rates were influenced by patient age and preoperative clinical conditions, but not by the type of hybrid graft.

However, the vast majority of patients required distal endovascular extension during follow-up.

Overall freedom from TEVAR at 5 years was 66% in the E-Vita group and 39% in the Thoraflex group.

In a competing risk analysis, patients with a Thoraflex prosthesis were significantly more likely to undergo TEVAR in the 5 years following the initial procedure. This is probably due to the rigid ring at the distal end of the stent graft of this prosthesis.

These results were not surprising and are similar to those recently reported by the Freiburg group. The authors reported a risk of reintervention of 31% at 1 year, 49% at 2 years and 64% at 3 years [19]. The same group of authors later studied the subset of patients treated with the trifurcated 100-mm stent graft implanted in arch zone 2. Downstream TEVAR was required on average 7 months after FET [20]. The most common indications were: diameter progression in 31 patients (47%), planned extension in 17 patients (26%) and dSINE in 12 patients (18%), while other rare reasons were also observed (kinking or rupture of the graft). However, all procedures were performed using a femoral approach and in-hospital outcomes were excellent, with no permanent SCI, stroke or death [20].

Finally, our results show that additional aortic reintervention may be necessary even after a second-stage treatment, underlining the importance of strict aortic follow-up. The ability to have an in-house endovascular programme facilitates the ability to offer a wide range of different solutions.

### Limitations

This study is retrospective and has all the potential drawbacks associated with such a study. However, the follow-up is very long considering the introduction of this technique in the early 2000s. In addition, we were able to compare a large number of the only 2 hybrid grafts available in the European market. We acknowledge that in some patients the E-Vita graft was preferred when a long stent was needed. This study provides valuable information on the outcomes following this procedure and highlights the need for further development of new hybrid grafts.

## CONCLUSIONS

Outcomes after FET are acceptable, especially the SCI rate has decreased significantly over the years, with the lowest rate reported for the Thoraflex graft. Although aortic reinterventions are very common, endovascular extensions are usually sufficient to control residual downstream aortic disease. Both hybrid grafts available in Europe have strengths and weaknesses and future challenges for new devices would be to facilitate proximal arch anastomosis, left subclavian reimplantation and late endovascular completions.

## Supplementary Material

ezad364_Supplementary_Data

## Data Availability

The data underlying this article will be shared on reasonable request to the corresponding author.

## Notes

Presented at the 36th EACTS Annual Meeting 2022, Milan, Italy.

## ABBREVIATIONS

AS: Aortic syndrome
AAS: Acute aortic syndrome
ASCP: Antegrade selective cerebral perfusion
CPB: Cardiopulmonary bypass
CSF: Cerebrospinal fluid
CT: Computed tomography
dSINE: Distal stent-induced new entry tear
FET: Frozen elephant trunk
SCI: Spinal cord injury
SD: Standard deviation
TEVAR: Thoracic endovascular aortic repair
