# Supplementary figures and images for: Frozen elephant trunk technique using hybrid grafts: 15-year outcomes from a single-centre experience
Source: Eur J Cardiothorac Surg. 2023 Oct 31;65(2):ezad364. doi: 10.1093/ejcts/ezad364 (PMC10859176; doi:10.1093/ejcts/ezad364)

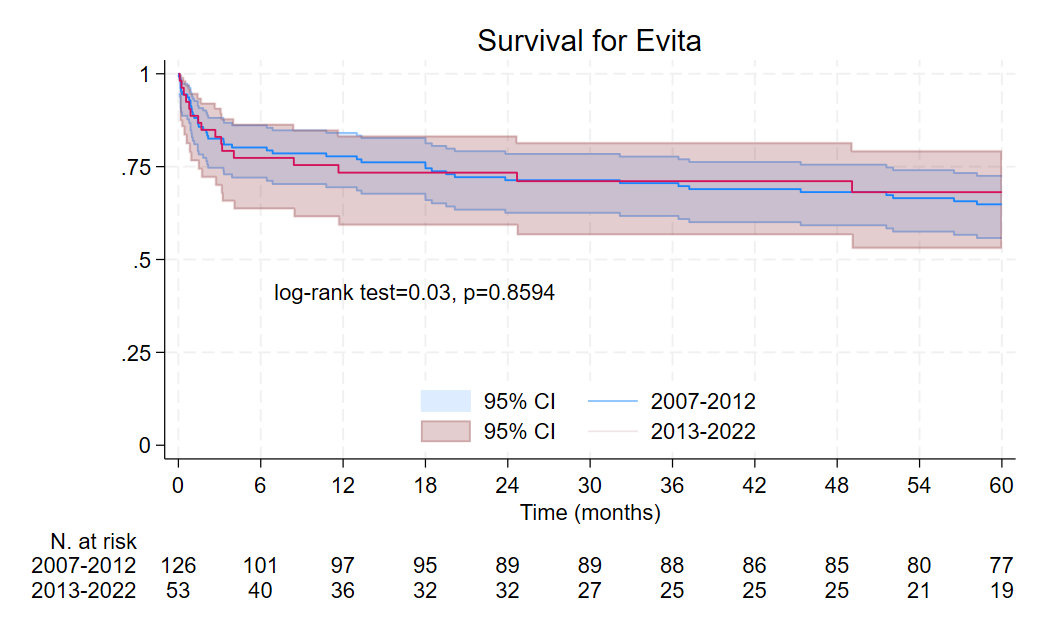

Supplement: ezad364_Supplementary_Data [file ezad364_supplementary_data.zip › Supplementary figure 1.tif]

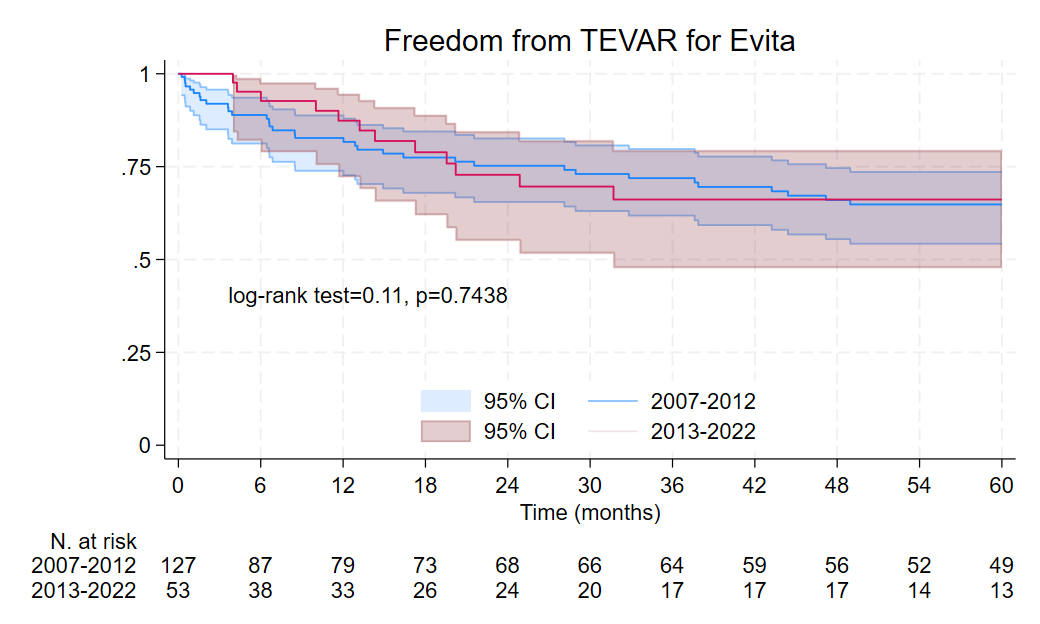

Supplement: ezad364_Supplementary_Data [file ezad364_supplementary_data.zip › Supplementary figure 2.tif]
